# Development and evaluation of a tailored mHealth parenting program for multicultural families: a three-arm cluster randomized controlled trial

**DOI:** 10.3389/fpubh.2023.1182310

**Published:** 2023-10-11

**Authors:** Hyunmi Son, Gyumin Han

**Affiliations:** College of Nursing, Research Institute of Nursing Science, Pusan National University, Yangsan-si, Gyeongsangnam-do, Republic of Korea

**Keywords:** mHealth, emigrants and immigrants, health behavior, health education, parenting

## Abstract

**Objective:**

Health management of children during early childhood requires substantial information. Multicultural families find it difficult to obtain and use parenting-relevant information for their young children. This study aimed to develop, implement, and evaluate a tailored Health parenting program and lay-health workers’ support to improve children’s health in multicultural families in Korea.

**Methods:**

In this study, we employed the Analysis, Design, Development, Implementation, and Evaluation (ADDIE) model as the conceptual framework, guiding the creation of a tailored mHealth application supplemented by a lay-health worker support module. The efficacy of the program was assessed through an experimental three-arm cluster randomized controlled trial. A total of 101 participants were stratified into three distinct groups as follows: (1) Experimental Group A, which received the mHealth program alongside the lay-health worker support component; (2) Experimental Group B, exposed solely to the mHealth program; and (3) Control Group, devoid of any intervention. Within these groups, 101 marriage migrant women hailing from Vietnam, the Philippines, and China were incorporated, with each group comprising 33, 30, and 38 participants, respectively. The study’s primary endpoint encompassed a comprehensive assessment of health-promoting behaviors, proficiency in eHealth literacy, and the family strength.

**Results:**

The analysis revealed noteworthy interactions among the three distinct groups over the course of time, with implications for health-promotion behaviors (*p* = 0.041), eHealth literacy (*p* = 0.037), and family strength (*p* = 0.044). Specifically, the experimental groups exhibited substantially elevated levels of the specified outcome variables when contrasted with the control group. Notably, the positive effects persisted even up to 12 weeks subsequent to the conclusion of the intervention, underscoring the program’s capacity to foster enduring improvements in the observed metrics.

**Conclusion:**

This study highlights the benefits of offering contextually appropriate information to target groups constrained by challenges in information access, evaluation, and utilization. Notably, drawing from their positive experiences in this process, we underscore the importance of employing lay health workers. These workers play a crucial role in fostering and ensuring sustained behavioral changes.

## 1. Introduction

In East and Southeast Asia, international marriages are on the rise and have substantial influence on the national populations (1). In South Korea, recent data show 173,882 such households in 2021, up from 87,964 in 2007, and the majority (about 80%) of immigrant spouses are women (2). Around 84% of these women become pregnant less than one year after marriage, and they also experience various physiological changes related to pregnancy, childbirth, and childrearing along with the stress of adapting to a new culture (3). Giving birth to and raising-children is a very important and difficult task for parents, but in the case of cultural differences and little support, such as marriage migrant women, the burden of child rearing increases considerably, impeding performance of the role of caregivers (4). Early childhood is the most active period of growth in life and thus is a decisive developmental period that forms the foundation for lifelong health; thus, parental education should be provided so that migrant women can fulfill their parental roles well.

Health management for children during early childhood requires substantial information. The parents are often placed in diverse situations, and having access to the necessary childcare information can enable them to respond more flexibly and appropriately (5). Most of the postpartum education about infant care is provided to new parents at or before hospital discharge. However, research shows that providing postpartum education only around the time of hospitalization reduces receptivity and recall (6). Research shows that new parents do not retain information that is only taught once (7), and they return to websites to recall information when in need (7, 8). Yet, the quality and reliability of information from digital sources vary substantially (9), and the information can be overwhelming and often causes anxiety among parents (10). Therefore, it is necessary for parents to develop eHealth literacy, which is the ability to find, understand, and apply appropriate online health information necessary for the management of their children’s healthcare.

Immigrants, specifically, often experience an even greater burden because they have to perform childcare in a new culture. It is reported that immigrant parents feel overwhelmed with a variety of challenges including the lack of family and community support, lack of access to linguistically appropriate services and resources, cultural conflict regarding parenting practices, fear related to social services, and language barriers (11). A large proportion of women that have immigrated to get married to Korean men (i.e., marriage immigrants) have low socioeconomic status and low formal education levels, tend to have low levels of health literacy to begin with, and find it more challenging to correctly evaluate and accept health information (12). Research also found that these women often experience family conflicts because their culture is different from that of their husbands (13). Thus, to facilitate immigrant mothers and their children’s effective navigation of the challenges, it is necessary to provide customized information about parenting that considers their special circumstances and contexts.

Mobile health (mHealth) interventions have been shown to be efficient in delivering tailored health-related information, which tends to be more effective than generic information at inducing behavioral change (14, 15). A tailored mHealth intervention can be designed to deliver essential health-related information tailored to the user’s characteristics. With the popularity of mobile technology, mHealth interventions are widely utilized because they are readily accessible without space and time constraints. They are also generally cost-efficient and effective for pediatric healthcare (14). Furthermore, in family-based interventions, if circumstances make it difficult for the whole family to meet directly, an mHealth intervention can be used to deliver accurate information to all family members (16). For marriage immigrant women, who often speak different languages than their husbands and their husbands’ families, mHealth intervention would be particularly advantageous.

While mHealth interventions offer numerous advantages, specific populations may require additional strategies to effectively replicate their effects. As many marriage immigrants may not be familiar with using mobile applications, implementing mHealth intervention alone may not be sufficient (16). Furthermore, the program contents may not be effectively delivered if the user does not perceive the intervention to be necessary or lacks the motivation to continue using it (17). To change the behaviors of individuals with a low socioeconomic status, providing relevant education through home visits is effective (18). For marriage immigrants—who lack access to medical services and health information due to linguistic and cultural differences and tend to have low level of health literacy in general—human support could increase the effectiveness of mHealth intervention in helping them to provide effective care and manage the health of their young children. Especially for individuals like marriage immigrants, the guidance of educated lay health workers who understand their unique circumstances is likely more effective than specialized medical professionals. Research shows that lay-health workers (LHWs) who meet with the study population on a regular basis to provide education and support can be effective for health interventions (19). Thus, our intervention combines an mHealth program and LHWs, who met with marriage immigrants on a regular basis to provide education and counseling. To investigate if the addition of LHWs indeed enhances the effectiveness of the mHealth intervention, this study includes an experimental group B that had the mHealth intervention alone without LHWs.

This study aimed to develop, implement, and evaluate a tailored mHealth program with and without LHWs on health-promoting behaviors, eHealth literacy, and family strength in multicultural families with young children. The main study hypotheses are as follows:

H1. There will be a significant increase in health-promoting behaviors among those who received the tailored mHealth intervention with and without LHW (mH and mH-L) compared to those who did not receive any intervention (control group).

H2. There will be a significant increase in eHealth literacy among those who received the mHealth intervention with and without LHW (mH and mH-L) compared to those who did not receive any intervention (control group).

H3. There will be a significant increase in family strength among those who received the mHealth intervention with and without LHW (mH and mH-L) compared to those who did not receive any intervention (control group).

## 2. Materials and methods

### 2.1. Study design

A mobile app-based parenting program for multicultural families with young children was developed and evaluated using the five stages of the ADDIE model: analysis, design, development, implementation, and evaluation (20). To test the effectiveness of the program, we used experimental design. Specifically, we used a cluster randomized controlled trial with three groups with a pretest (T0), post-test (T1) immediately after the intervention during Week 8, and follow-up during Week 20 (T2).

The duration of the intervention was eight weeks for all participants. The experimental group (mH-L) received eight weeks of mobile application use and LHW support. The LHWs’ activity consisted of approximately twenty-five minutes of support and counseling during each visit, and they provided positive reinforcement for participants who were comfortable using the application. The experimental group B (mH) received only the mobile application without LHW support. The control group was observed without either a mobile application or LHW support.

Given that marriage immigrant women who move to Korea often use a multicultural family support center near their home, there is a concern that potential interactions at the support centers may affect the study outcome. To address this concern, we used a cluster randomization method to randomly assign centers to the three study groups.

### 2.2. Participants

Study participants were women who immigrated to Korea from China, the Philippines, and Vietnam to marry Korean men and who currently have children aged 0–6. We recruited participants who had visited Multicultural Family Support Center (MFSC). To be eligible to participate in the study, women had to own an Android smartphone and have the ability to download and use the mobile application. The study participants then had to agree to not participate in another early-childhood healthcare program during the intervention period.

**Figure 1:**
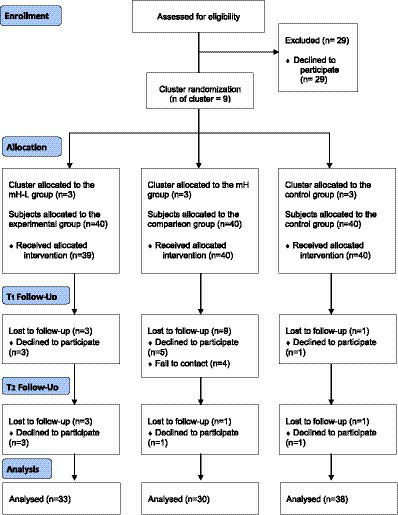
Recruitment of the program.

Since social welfare services for marriage migrant women who move to Korea are mainly delivered through MFSC, most marriage migrant women use the MFSC. Therefore, this study recruited subjects through the MFSC. There are thirty-eight MFSC in three adjacent metropolitan cities, and we used the RANDBETWEEN function in Microsoft Excel 2010 to randomly assign three centers for each of the three study groups. According to the power calculation using G-power 3.1.9.2, the minimum number of participants needed was 54 women, 18 individuals in each group. However, we considered that a similar study conducted in the past had the dropout rate of 40% (21), and, given that the current study utilized repeated measures, we recruited a total of 120 women, 40 for each group. The actual number of participants in the final analysis were 101 women, 33 in the mH-L group, 30 in the mH group, and 38 in the control group (Figure 1).

**Table 1:** Process of program development base on ADDIE model.

Stage	Contents	Details
Analysis	Literature review & Contents analysis	Literature analysis of health promoting behaviors, eHealth literacy, and family strength
		Health communication strategies
	Target population analysis	Analysis of experiences of parenting (marriage immigrant women, Korean husbands, mother-in-law)
		Analysis of multicultural family dynamics
		Analysis of factors affecting parenting behaviors
		Analysis of factors affecting eHealth literacy
		Needs analysis in online health information
	Environment analysis	Immigrant support specialist focus group interviews and Delphi study
		Analysis of websites & apps related to child health
Design	Establish the goals and purposes of the program	mHealth program topic/content selection and education design
	Develop contents and format of the preliminary program	Development of mobile application UI scenarios to provide customized health information
	Plan research study evaluating the effects of the program	A three-arm cluster randomized controlled trial
Development	Develop detailed contents and strategies	Tailoring for subjects’ mother tongue and child’s age
		Lay health workers’ support
	Evaluate the preliminary program	Expert review and validity evaluation
		Pilot test and modification of the program
Implementation	Implement the program for 8 weeks	mH-L group: tailored mHealth program and support of lay health workers mH group: tailored mHealth program Control gorup: user care
Evaluation	Evaluate the effects of the program	Outcome variables: health promoting behavior, eHealth literacy, family strength Measure time: before intervention (T0), Week 8 (T1). Week 20 (T2)

### 2.3. Intervention

#### 2.3.1. mHealth application

A mobile app-based parenting program for multicultural families with young children was developed using the five stages of the ADDIE model: analysis, design, development, implementation, and evaluation (20). Table 1 provides a succinct summary of each stage of the ADDIE.

**Table 2:** Characteristics of the mHealth program participants (*N* = 101).

Characteristics	Categories	n(%) or M ± SD			χ^2^/F	*p*
		mH-L (*n* = 33)	mH (*n* = 30)	Cont. (*n* = 38)		
Age (yr)		30.12 ± 14.05	29.10 ± 5.85	28.87 ± 4.70	0.205	0.815
Country of origin	Vietnam	21 (63.6)	20 (66.7)	31 (81.6)	3.865	0.421
	China	9 (27.3)	7 (23.3)	6 (15.8)		
	Philippines	3 (9.1)	3 (10.0)	1 (2.6)		
Education level	Elementary school or less	2 (6.1)	3 (10.0)	1 (2.6)	3.922	0.708
	Middle school	10 (30.3)	12 (40.0)	11 (28.9)		
	High school	15 (45.5)	9 (30.0)	16 (42.1)		
	College and above	6 (18.2)	6 (20.0)	10 (26.3)		
Job status	Unemployed	26 (78.8)	21 (70.0)	31 (81.6)	1.346	0.576
	Employed	7 (21.2)	9 (30.0)	7 (18.4)		
Duration of stay in Korea (yr)		4.16 ± 2.51	4.06 ± 2.57	5.20 ± 2.48	2.227	0.113
Duration of marriage (yr)		4.25 ± 2.55	4.16 ± 2.00	5.13 ± 2.17	1.918	0.152
Korean language ability		2.79 ± 0.76	2.89 ± 0.73	3.09 ± 0.57	1.814	0.169
Primary caregiver	Self	15 (45.5)	15 (50.0)	16 (42.1)	2.874	0.593
	Father	2 (6.1)	1 (3.3)	0 (0.0)		
	Both	16 (48.5)	14 (46.7)	22 (57.9)		
Parenting assistant	None	7 (21.2)	15 (50.0)	12 (31.6)	14.870	0.051
	family in law	15 (45.5)	10 (33.3)	21 (55.3)		
	Family on the maternal side	9 (27.3)	2 (6.7)	3 (7.9)		
	Neighbor	1 (3.0)	2 (6.7)	0 (0.0)		
	Etc.	1 (3.0)	1 (3.3)	2 (5.3)		
Number of children	1	20 (60.6)	24 (80.0)	22 (57.9)	4.104	0.143
	≧ 2	13 (39.4)	6 (20.0)	16 (42.1)		
Perceived child’ health status		4.12 ± 0.78	4.00 ± 0.79	4.03 ± 0.68	0.237	0.789
Family Affluence		7.39 ± 1.77	6.93 ± 1.72	6.95 ± 1.63	0.787	0.458

First, for the analysis stage, we conducted a systematic review of the research literature on early childhood health care programs and analyzed known intervention strategies applicable to immigrants, educational characteristics, and effects. In addition, to maximize the impact of the intervention, the participants and the surrounding environment were also carefully analyzed. These analyses revealed that marriage immigrant women experience communication difficulties and family conflicts due to cultural and language differences, and such family health issues affected children’s health-related behaviors. We also found that, while these women wanted to find health-related information online, they often had difficulty finding, selecting, and utilizing reliable information. In the expert analysis, we learned that these women often lack motivation to improve parenting quality and did not know how to effectively resolve family conflicts. The intervention program needed to address these areas.

Second, for design and development stages, we designed the contents of the program to include information on growth and development, disease management, healthy living, vaccination, and nutrition, all based on the results of the above analysis and input from the participants and experts. First, we created user interface (UI) scenarios to develop the mobile application for marriage immigrant women to help them with parenting and improving the health of infants and young children in their families. An algorithm providing tailored health information for each registered child’s age was developed, and the health information provision flow was written as a UI scenario. The UI scenario according to personalized information regarding their child’s age and language was used to tailor the content of the mobile application.

The application was developed by discussing the content to be implemented through the UI with two developers who have experience in developing multiple apps. A pilot test to assess the efficacy and safety of the developed mobile application was conducted on eight marriage immigrant women who had experience raising young children. After the participants had operated the mobile application for one week, we assessed factors such as stability of use, content readability, satisfaction with the application, and the ease and utility of information delivery. Based on the results, the mobile application was modified and improved to produce the final version.

**Figure 2:**
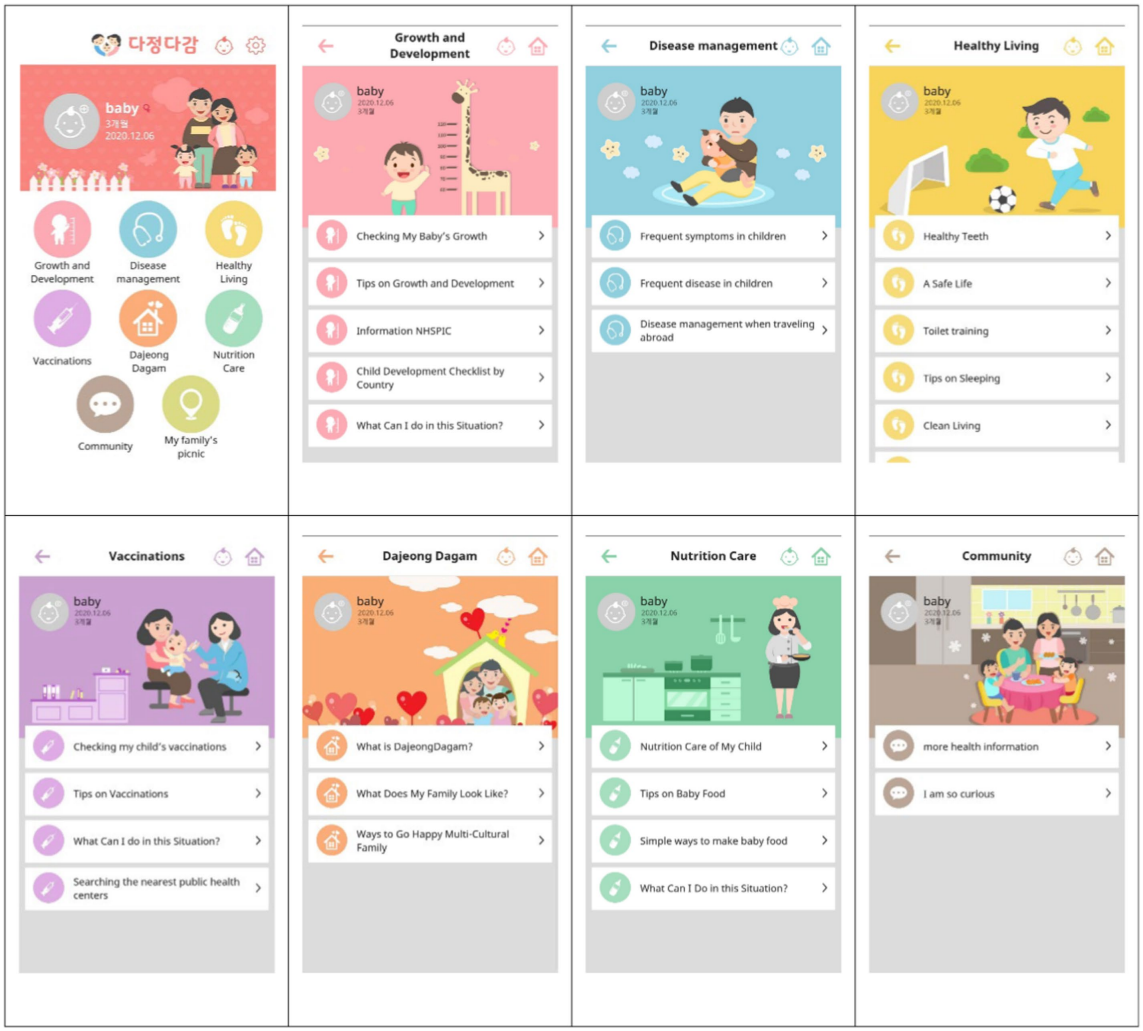
Screenshots of DaDa.

The final mobile application was named “DajeongDagam” (hereafter, DaDa), which is a Korean abbreviation for “coming closer to multicultural families” (see Figure 2). The content of DaDa consists of essential health information related to young children, including the following issues: growth and development, disease management, healthy lifestyle (e.g., dental hygiene, safety, potty training, sleep management, personal hygiene, and nursery), vaccination, and nutritional management. To reinforce family strength, animations were provided that illustrated problems that may occur due to cultural differences as well as potential solutions. To account for linguistic differences between the participants, DaDa underwent translation and editing for Korean, English, Chinese, and Vietnamese by a translation team consisting of migrant women who were fluent in Korean as well as their native language. The translation of the content was commissioned to a professional translation agency, and the organization, an interpreter and translation cooperative established by an organization supporting migrant women, tried to improve the validity of the translation by reviewing the translated content by migrant women fluent in Korean and their mother tongue.

Importantly, the information was constructed to provide a tailored experience, depending on the age of the child (in months). To provide personalized information, the participants provided their IDs, passwords, and countries of origin and their child’s date of birth. Based on these details, the participants were provided with information tailored to their child’s age in the language of their country of origin. To change participants’ behavior, the content of DaDa was not limited to information on childcare but also included the ability to log the child’s biological changes as well as providing instructional images and videos. Simple terminology was used to aid participants’ understanding, and a message board was established that allowed participants to share their personal experiences and information related to the care of young children. If the participants had a question, they could ask it through the Q&A message board in the mobile application, and an expert would respond within three days. We used the push function to motivate the target user to engage with the app. Content designed to capture the user’s attention was displayed, and upon clicking, it led them directly into the app.

#### 2.3.2. Lay-health workers

The LHWs provided counseling and support through home visits during an eight-week intervention period. A total of twelve LHWs were involved in the study, and they consisted of staff members who directly provided support services to multicultural families at the MFSC, meaning that they had frequent contact with multicultural families. Any staff member selected to be a LHW had to have at least one year of experience providing support services to multicultural families.

Before the program began, the LHWs participated in two four-hour sessions. The training introduced and educated them about the application’s educational content, the method of use, and the content required for counseling. In addition, a manual containing tips for support and counseling as well as instructions on how to use the mobile application and contents were provided to LHWs for continued use.

The LHWs visited participants’ homes during the intervention period and provided counseling in case the participant had any difficulties using the application or had questions about healthcare information concerning their young children. They also introduced the participants to the expert Q&A through which they could ask questions and provided support for them to continue using the mobile application.

A total of 12 LHW participated in this study, and the researcher confirmed their activities and kept a journal so that support could be provided as intended. In addition, we kept in close contact with them to continuously manage problems that occurred during support. The content of their activities consisted of program usage guide, explanation and recommendation to use the program, problem identification, and connection to additional services. The LHWs’ activity consisted of approximately thirty minutes of support and counseling during each visit.

### 2.4. Data collection

Data were collected between September 18, 2017, and June 30, 2018. The mH-L, mH, and control groups each completed a pretest survey (T0), two post-test surveys at T1 (Week 8), and a follow-up test at T2 (Week 20). The method for data collection was a paper-based self-administered questionnaire survey.

The research instruments consisted of structured questionnaires, used with permission from the original developers and translators. The questionnaires included sociodemographic characteristics, healthcare behaviors for young children, eHealth literacy, and family strength. The instruments utilized Korean, English, Chinese, and Vietnamese translations, meaning that participants could either select the Korean version or that in their native language. The instruments were translated by a professional organization, which was a translation and interpretation cooperative founded by a group supporting immigrant women. The instruments were translated by immigrant women who were fluent in Korean and their native language, and the translated content was further edited to improve its validity.

### 2.5. Variables

As a result of the program, to verify whether marriage migrant women effectively manage their children’s health, the primary outcome was measured as marriage migrant women’s children’s health-promoting behavior. We adapted the early childhood health-promoting behavior instrument developed by Kim et al. (22). The instrument measures healthcare behaviors based on seven factors: safety, emotional support/endeavor, activity/rest, disease prevention, appropriate clothing, nutrition, and cleanliness/hygiene. Each item was scored on a 4-point Likert scale with higher scores indicating better performance of early childhood healthcare behaviors. We modified some of the questions to fit the objectives of the present study, and two nursing professors evaluated the modified items to ensure the content validity. The Cronbach’s alpha values of health-promoting behavior were 0.950 for T0, 0.967 for T1, and 0.957 for T2.

To measure eHealth literacy, we utilized the e-HEALS scale by Norman and Skinner (23), and the instrument was modified to fit the subject of healthcare regarding young children. Two nursing professors checked the content. There were eight questions in total, and each of them was scored on a 5-point Likert scale. The Cronbach’s alpha values of eHealth literacy were 0.941 for T0, 0.928 for T1, and 0.965 for T2.

To measure family strength, we used the family strengths and capabilities instrument developed by Deal et al. (24) and validated by Danışman et al. (25). This instrument consists of 26 questions scored on a 5-point Likert scale. The Cronbach’s alpha values of family strength were 0.962 for T0, 0.975 for T1, and 0.968 for T2.

The content validity of the instruments used in the user experience of the mobile application was checked in advance by two nursing professors. The reliability of the instruments used in the pilot was found to be always 0.8 or higher, indicating an acceptable level of reliability.

### 2.6. Ethical considerations

The study was approved beforehand by the Institutional Review Board at our institution (OOO IRB/OOOO_OO_OO). To ensure the autonomy of participants, the study’s aims and methods were thoroughly explained prior to their participation. Participants with a low Korean-language aptitude were given the explanations in their native language through a translation/interpretation expert; the consent form was also written in their native language. Lastly, as an additional ethical consideration, after the mH-L and mH groups had completed the intervention program, the control group was also given access to the mHealth application along with an approximately fifteen-minute orientation on how to use it.

### 2.7. Data analysis

The collected data were analyzed using SPSS WIN 25.0, and a two-tailed test was performed at the significance level of 0.05. The normality test of the dependent variable was verified using the Kolmogorov–Smirnov test, and it was found that it was not normally distributed. This study is designed to compare the effects of interventions with repeated measurements, so repeated measures analysis of variance is generally considered, but the normality of the dependent variable is not satisfied and the repeated measures analysis of variance is not appropriate. In consideration of this, the generalized estimating equation (GEE), which is an extension of the generalized linear model, was analyzed. The generalized estimation equation is a method of applying multiple regression analyzes in consideration of the intra-subject correlation of measured values. It can be applied even if the assumption of normality is not satisfied, and it can be analyzed in data containing missing values by considering time as a variable.

Descriptive statistics were employed to summarize the general characteristics of the subjects, and the comparability of the three groups was assessed using χ2-test, Fisher’s exact test, or ANOVA, as appropriate. Prior to the intervention, an ANOVA homogeneity test was performed to examine the equality of variance in the dependent variables across the three groups. Disparities among the three groups concerning the time elapsed before and after the implementation of the intervention program were examined utilizing GEE. To assess the distinctions among the mH-L, mH, and control groups at time points T1 and T2, we conducted the Kruskal-Wallis test. Subsequently, a Mann–Whitney test was employed as a *post hoc* analysis for the three groups. A significance level of 0.05 was initially set, which was adjusted to 0.017 using the Bonferroni correction.

## 3. Results

### 3.1. General characteristics and homogeneity test of subjects

Table 2 presents both the demographic characteristics and the outcomes of the homogeneity tests conducted across the three groups. The mean age of participants was 30.12 years for the mH-L group, 29.10 years for the mH group, and 28.87 years for the control group. In terms of nationality, 63.6% of the mH-L group, 66.7% of the mH group, and 81.6% of the control group hailed from Vietnam. Regarding educational level, middle school graduates accounted for 30.3, 40.0, and 28.9% of the mH-L, mH, and control groups, respectively. Regarding employment status, percentages of unemployment were noted as 78.8% for the mH-L group, 70.0% for the mH group, and 81.6% for the control group. The duration of residence in Korea was found to be 4.16 years for the mH-L group, 4.06 years for the mH group, and 5.20 years for the control group. Additionally, the duration of marriage was observed to be slightly longer than the period of residence in Korea, with respective values of 4.25 years for the mH-L group, 4.16 years for the mH group, and 5.13 years for the control group. Furthermore, when asked about their child’s primary caregiver, 45.5, 50.5, and 42.1% of participants in the mH-L, mH, and control groups, respectively, indicated that the caregiver was solely responsible. Family affluence, assessed as an indicator of household wealth, yielded scores of 7.39, 6.93, and 6.95 for the mH-L, mH, and control groups, respectively. Upon examining the homogeneity of these general characteristics among the three groups, no statistically significant differences were observed across all parameters (*p* > 0.05), affirming the comparability of the groups prior to intervention.

### 3.2. Pretest homogeneity for dependent variables

First, Table 3 shows the results of the pre-intervention homogeneity testing for the dependent variables. Results show that participants in the mH-L, mH, and control groups did not differ significantly in terms of the three dependent variables of health-promotion behaviors, eHealth literacy, and family strength.

**Table 3:** The program’s effect.

Variables	Time	mH-L^a^	mH^b^	Control^c^	Source	χ^2^	*p*	*Post hoc*
		M ± SE	M ± SE	M ± SE				
Health-promoting Behavior	Pre	3.37 ± 0.08	3.49 ± 0.08	3.44 ± 0.06	Group	0.732	0.694	–
	T1	3.55 ± 0.08	3.60 ± 0.07	3.52 ± 0.06	Time	16.905	<0.001	–
	T2	3.69 ± 0.06	3.57 ± 0.08	3.51 ± 0.05	Group *Time	9.951	0.041	a > c
eHealth Literacy	Pre	3.51 ± 0.16	3.70 ± 0.12	3.51 ± 0.13	Group	10.314	0.006	–
	T1	3.88 ± 0.11	4.13 ± 0.13	3.64 ± 0.09	Time	13.125	0.001	b > c
	T2	4.12 ± 0.13	4.05 ± 0.14	3.50 ± 0.11	Group *Time	10.206	0.037	a,b > c
Family Strength	Pre	4.11 ± 0.12	4.18 ± 0.12	4.05 ± 0.10	Group	4.989	0.083	–
	T1	4.20 ± 0.10	4.51 ± 0.09	4.17 ± 0.09	Time	8.567	0.014	b > c
	T2	4.40 ± 0.09	4.37 ± 0.09	4.13 ± 0.09	Group *Time	9.806	0.044	–

### 3.3. Program’s effect

**Figure 3:**
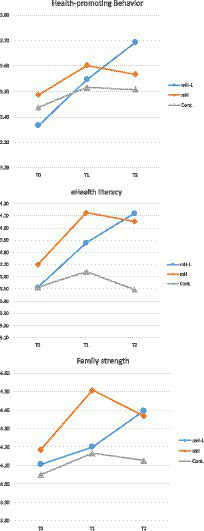
Changes of dependent variables by time.

The effects of the tailored mHealth program and the LHWs on three study dependent variables concerning early childhood healthcare behaviors are presented in Table 3 and Figure 3.

#### 3.3.1. Health-promotion behaviors

The first hypothesis that there would be a significant increase in health-promoting behaviors among those who received the mHealth intervention with LHW (mH-L group) compared to those who did not (control group) was supported by the data. The mH-L group demonstrated a steady increase in health-promoting behaviors at three points (M = 3.37, 3.55, and 3.69) with significantly higher levels at T2 compared to the control group (M = 3.51). Data also show that, while mH group had some increase in health-promoting behavior at T1 (M = 3.60), the increase was not sustained at T2 (M = 3.57). The interactions between the three groups and over time indicated statistically significant differences in health-promoting behaviors (χ^2^ = 9.951, *p* = 0.041).

The examination of group distinctions at the T1 time point revealed no statistically significant differences among the three groups (H = 1.375, *p* = 0.503). However, a statistically significant distinction was observed among the three groups at the T2 time point (H = 7.832, *p* = 0.020). *Post hoc* analysis results indicated that the mH-L and mH groups (U = 392.000, *p* = 0.150), as well as the mH and control group (U = 463.500, *p* = 0.188), did not exhibit statistically significant differences. In contrast, a statistically significant difference was identified between the mH-L and control groups (U = 388.500, *p* = 0.006).

#### 3.3.2. eHealth literacy

The second hypothesis that there would be a significant increase in eHealth literacy among those who received the mHealth intervention with LHW (mH-L group) compared to those who did not (control group) was also supported by the data. As in the case of health-promoting behaviors, the mH-L group demonstrated a steady increase in eHealth literacy over time (M = 3.51 to 3.88 and then to 4.12). At T2, the mean eHealth literacy for the mH-L group was significantly higher than for the control group (M = 3.50). Analysis revealed a statistically significant interaction in eHealth literacy over time among the three groups (χ2 = 10.206, *p* = 0.037). As with the health-promoting behaviors, the mH group showed an increase in eHealth literacy immediately after the intervention (M = 4.13), which decreased slightly (M = 4.05) at T2.

The examination of eHealth literacy differences among the three groups at T1 and T2 yielded statistically significant results (T1; H = 7.960, *p* = 0.019, T2; H = 14.536, *p* < 0.001). Subsequent *post hoc* analyses at T1 indicated no statistically significant differences between the mH-L and mH groups (U = 395.000, *p* = 0.167), as well as between the mH-L group and the control group (U = 493.000, *p* = 0.119). However, a statistically significant difference was observed between the mH group and the control group (U = 350.500, *p* = 0.006). At T2, *post hoc* analyses showed no statistically significant difference between the mH-L and mH groups (U = 475.500, *p* = 0.786). Conversely, statistically significant differences were observed between the mH-L group and the control group (U = 333.000, *p* < 0.001), as well as between the mH group and the control group (U = 326.000, *p* = 0.002).

#### 3.3.3. Family strength

The final hypothesis that there would be a significant increase in family strength among those who received the mHealth intervention with LHW (mH-L) compared to those who did not (control group) was supported by the data as well. As with the two other dependent variables, family strength increased over time among the mH-L group (M = 4.11, 4.20, and 4.40). At T2, the mean family strength score for the mH-L group was significantly higher than that for the control group (M = 4.13). The interactions among the groups and over time indicated statistical significance in family strength (χ2 = 9.806, *p* = 0.044). It is worth noting that the mean family strength score for the mH group was 4.51, even higher than that of the mH-L group (M = 4.20), but it declined substantially at T2 to levels (M = 4.37) lower than that of the mH-L group (M = 4.40).

Upon analyzing the disparities in family strength among the three groups at both T1 and T2, significant differences emerged (T1; H = 7.051, *p* = 0.029, T2; H = 6.224, *p* = 0.045). *Post hoc* analysis at T1 indicated the absence of statistically significant distinctions between the mH-L and mH groups (U = 352.500, *p* = 0.049), as well as between the mH-L group and the control group (U = 592.500, *p* = 0.690). Nonetheless, a notable statistically significant difference was identified between the mH group and the control group (U = 361.000, *p* = 0.010). In contrast, no significant differences were observed at the T2 time point for comparisons between mH-L and mH (U = 470.000, *p* = 0.730), mH-L and the control group (U = 430.000, *p* = 0.023), and mH and the control group (U = 414.500, *p* = 0.055).

## 4. Discussion

This study aimed to develop a comprehensive and tailored mHealth intervention program that promotes healthy behaviors for children from multicultural families. The program involved Lay Health Worker (LHW) elements that were incorporated to optimize the effectiveness of the mHealth intervention based on participants’ characteristics. There is a substantial need to provide support for marriage immigrant women to help improve their parenting and manage health issues of their young children better. Learning to promote and manage necessary health care for young children is a critical public health matter in Korea. This is a rare study that developed and tested an mHealth application specifically designed to help marriage immigrant women. It demonstrated that a tailored mHealth program, combined with an LHW component, can effectively contribute to enhanced health-promoting behaviors, eHealth literacy, and family strength in multicultural families. This program is meaningful because it directly improved families’ health behavior. These findings are in line with Conway et al.’s (15) general effectiveness of tailored mHealth interventions. The result is the outcome of increased health-related behaviors as tailored information that considers the characteristics of individuals motivates behavior changes. These results suggest that using mHealth would work effectively in providing tailored information. Delivering tailored information using a mobile phone or computer is more effective because it can utilize materials such as videos and pictures (26). Therefore, tailoring intervention using mHealth is an effective strategy to change the subject’s health behavior.

This study also addressed the relative importance of the LHW component. Specifically, within the mH-L group, supported by LHW, children’s healthcare behaviors and eHealth literacy at T2 surpassed those of the control group. These findings imply an impact on sustained behavioral changes compared to the group solely receiving mHealth interventions. This aligns partially with prior research indicating the efficacy of LHW in improving behavior among participants with lower socioeconomic status (18). Challenges of mHealth programs targeting vulnerable populations encompass limited accessibility and technical obstacles (17). In this study, LHW was engaged to assist women in installing and navigating mHealth applications, resolving issues, and promoting application utilization. Through these avenues, LHW may have contributed to enhancing the program’s long-term effectiveness by aiding women in overcoming technical challenges and cultivating self-efficacy through successful parenting behaviors (27). Thus, for individuals with low eHealth literacy who struggle to access, comprehend, evaluate, and effectively apply information to foster the healthy upbringing of their children, strategies involving human resources to enhance actual behaviors become imperative, rather than relying solely on standalone mHealth interventions.

The mH group, which received the mHealth program, showed an improvement in eHealth literacy after the intervention. The mH-L group showed a sustained increase. eHealth literacy refers to everything from finding, evaluating, and applying electronic information to solving health problems (23). It includes not just obtaining information but also having the capability to use it. In this study, the information provided on child rearing was verified by experts, thereby ensuring that the individuals could trust the information given to them. The information was classified according to the age of the child so that necessary details could be identified and applied immediately. The success of this intervention in terms of information utilization resulted in an improvement in eHealth literacy. In particular, the participants with LHWs had additional support to ensure accurate utilization of the health information. Since finding, understanding, and utilizing health information is an important factor in behavior change, as opposed to merely being informed (28), improvement in eHealth literacy is particularly crucial. Additionally, these results have significant implications, because they mean that marriage immigrant women can critically select, evaluate, and apply online information in the current era where a lot of information is being distributed online.

The mH-L and mH groups, both of which used the mobile application, showed improved family strength. Marriage immigrant women, having moved to a new society, are dependent on their husbands (13). Specifically, when the mother applies different childcare methods than her husband or mother-in-law, who exert greater authority in these matters, family conflict regarding childcare issues is inevitable (3, 13). In such situations, education though animation is an effective way to aid the participant’s understanding (29). This study provided information in the participants’ native language, which could easily be converted into Korean so that information about children could be shared with other families. The mHealth program in this study provided case studies in the form of animations to resolve family conflicts resulting from cultural differences. These are considered to improve family health by enhancing mutual cultural understanding (30). Thus, multicultural families who are raising a child in an environment where different childcare methods may cause conflicts, support is needed for adapting to the dual cultures.

The ability to observe these effects in this study stemmed from a rigorous analysis of participants and their surrounding environment based on the ADDIE model. In the analysis stage of the ADDIE model, the participant and the environment surrounding the participant were thoroughly analyzed to deliver tailored health information to the target population. Looking at the existing parent support program for immigrants, it was found that most studies considered tailoring programs for cultural adaptation (31). However, by analyzing the participants and their surrounding environment, we found that there were difficulties in language, family conflicts, and application of information when parenting children. Thus, essential information for each age was provided to generate information about the growth and development of children, and the app was configured to check the previous age group, if desired. In addition, animation was composed to resolve family conflicts caused by different cultures. The provision of tailored information from such thorough analysis was quite effective in delivering a large amount of information and increasing knowledge and skills for better parenting among these women. Therefore, it is desirable to apply user-customized mHealth information for parenting. Additionally, considering the global situation where there are only a few opportunities such as the COVID-19 crisis to face the target population directly, tailoring mHealth intervention is meaningful as it can be used as an effective strategy.

Our results support the conclusion that, while mHealth interventions offer advantages such as the lack of spatiotemporal restrictions and the ease of access to relevant information and services, addition of interpersonal support component such as LHWs may be necessary to ensure lasting program effectiveness. This is especially important for programs directed at vulnerable populations like marriage immigrant women.

### 4.1. Limitations

First, our study only included participants who already owned an Android smartphone and had the ability to download and use the mobile application. Those who did not own their own phone or lacked the ability to work with mobile applications were excluded from the study. Additionally, we were unable to verify the actual usage time of the mobile application. As such, our findings may not be generalizable to women who have a lower level of technology competency or who lack access to smartphones. This may be particularly problematic when our findings are generalized to people in regions where the level of smartphone penetration or technological environment may differ substantially. Second, since the participants in this study were marriage immigrant women from China, Vietnam, or the Philippines, our program only addressed the childcare cultures of these countries. We did not account for the cultural characteristics of other countries—for instance, those outside of Southeast Asia—and generalizing the study results beyond these countries should be done with caution. Third, due to the nature of the intervention, the LHWs and participants were not blinded to the treatment condition they were assigned to. Fourth, family strength was only measured from the mother’s perspective, and we were not able to get data from other family members. Thus, the variable family strength is a subjective assessment of the women about the family and may differ from those of other family members.

## 5. Conclusion

The “DajeongDagam,” a tailored mHealth parenting program to improve early childhood healthcare, effectively improved health-promoting behaviors, eHealth literacy, and family strength among marriage immigrant women. Moreover, we also confirmed that using LHWs for additional interpersonal support for utilizing the mHealth application produced sustained behavioral changes well beyond the conclusion of the intervention. Future studies are needed to further enhance our understanding about mHealth interventions directed at vulnerable populations. In concluding this study, several pivotal insights emerged. Firstly, the significance of a comprehensive and meticulous examination of the target population cannot be overstated. For interventions aimed at vulnerable groups, such as married immigrants, understanding the distinct characteristics and challenges of this demographic is crucial. Our rigorous analysis enabled the creation of finely tailored messages, thereby enhancing their effectiveness. Secondly, technological interventions, notably eHealth and mHealth platforms, while promising, demand caution in deployment among vulnerable populations. Absent immediate supporting mechanisms — exemplified by Lay Health Workers (LHWs) in our scenario — the effectiveness of such programs can be jeopardized. In light of these findings, it becomes evident that to ensure sustained behavioral changes post-intervention, continued interpersonal resources are indispensable. This ensures the longevity of positive outcomes, cementing the initial benefits introduced by the program.

## Data availability statement

The original contributions presented in the study are included in the article/supplementary material, further inquiries can be directed to the corresponding author.

## Ethics statement

The studies involving humans were approved by Pusan National University Institutional Review Board (PNU IRB/2017_90_HR). The studies were conducted in accordance with the local legislation and institutional requirements. The participants provided their written informed consent to participate in this study.

## Author contributions

HS and GH: conceptualization, data curation, investigation, supervision, methodology, project administration, resources, software, validation, visualization, and writing—review and editing. HS: funding acquisition and writing—original draft. All authors contributed to the article and approved the submitted version.
